# Sexual History Taking in Acute Psychiatric Inpatient Admissions: An Audit of Documentation and Clinician Practice

**DOI:** 10.1192/bjo.2026.11560

**Published:** 2026-06-30

**Authors:** Rahat Ali, Antonio Lopes, Farah Ahmed, Deniz Ceylan

**Affiliations:** 1 Devon Partnership NHS Trust, Torquay, United Kingdom; 2 CENTRAL AND NORTH WEST LONDON NHS FOUNDATION TRUST, London, United Kingdom; 3 TORBAY AND SOUTH DEVON NHS FOUNDATION TRUST, Torquay, United Kingdom

## Abstract

**Aims:**

Background:

Sexual safety is a core component of providing a safe and therapeutic inpatient mental health environment. Devon Partnership NHS Trust's (DPT) Sexual Safety Policy (C60) outlines responsibilities to protect patients from sexual harm, promote consent, and ensure appropriate sexual health assessment. National guidance from NICE, the CQC, and the Sexual Safety Collaborative highlights the importance of routine sexual history taking, trauma-informed care, and safeguarding, particularly for vulnerable inpatient populations.

Aim: This audit aimed to evaluate compliance with DPT's Sexual Safety Policy (C60) on Haytor Ward by assessing documentation of sexual health and sexual safety during inpatient admissions, and to explore resident doctors’ knowledge, attitudes, and practices regarding sexual history taking.

**Methods:**

A retrospective review of ten inpatients admitted to Haytor Ward in May 2025 was conducted using System One (s1). Patients were selected using a randomised bed-number approach. Documentation within physical health clerking, the assessment tab, and tabbed journal entries was reviewed using predefined search terms aligned with Trust policy requirements. In addition, a qualitative and quantitative survey of South Devon resident doctors was undertaken using Google Surveys to assess awareness of policy, clinical practice, and perceived barriers. The survey was open for 30 days and achieved a 50% response rate.

**Results:**

Documentation of sexual health and sexual safety was below expected Trust standards. Sexual history was inconsistently recorded, largely due to the absence of a sexual health section within the routinely used admission clerking proforma. The existing Sexual Health template on s1 was not easily accessible and did not capture several policy-required elements, including gender identity, sexual orientation, sexual safety understanding, historyof sexual harm, and MAPPA/safeguarding status. Patients who were acutely unwell on admission were less likely to engage in sexual history discussions. Only 50% of surveyed doctors were aware of the Sexual Safety Policy, and most reported not routinely undertaking sexual history taking, citing time pressures, patient distress, lack of training, and system-level barriers.

**Conclusion:**

Sexual safety assessment on Haytor Ward is inconsistently documented, primarily due to IT system limitations and reduced staff awareness. Improving S1 templates, enhancing training, and embedding sexual safety into routine admission processes are essential to align practice with Trust policy and national guidance, and to ensure patient dignity, safety, and safeguarding.

